# Criteria A and B of the Alternative DSM-5 Model for Personality Disorders (AMPD) Capture Borderline Personality Features Among Adolescents

**DOI:** 10.3389/fpsyt.2022.828301

**Published:** 2022-04-11

**Authors:** Rasa Barkauskienė, Elena Gaudiešiūtė, Asta Adler, Lina Gervinskaitė-Paulaitienė, Alfredas Laurinavičius, Gabrielė Skabeikytė-Norkienė

**Affiliations:** Institute of Psychology, Vilnius University, Vilnius, Lithuania

**Keywords:** level of personality functioning, maladaptive personality traits, Alternative Model for Personality Disorders (AMPD), LoPF-Q 12–18, borderline personality features, adolescence, ICD-11

## Abstract

The recent development of a dimensional view toward personality disorder opens up the field of personality research based on the constructs of personality functioning (Criterion A) and maladaptive personality traits (Criterion B) as core components of personality pathology. However, little is known about the roles of these aspects in relation to borderline personality features during adolescence. The current study aimed at exploring the associations of Criterion A and B and their contribution in predicting borderline personality features in adolescence. A sample of 568 adolescents aged 11–17 (*M* = 14.38, *SD* = 1.57; 42.4% males) from different backgrounds (community-based, psychiatric inpatients, and youth forensic care) completed a set of questionnaires among which were measures of personality functioning, maladaptive personality traits, and borderline personality features. The findings reveal that Criterion A and B are strongly interrelated and both are significant in predicting borderline personality features in adolescents. Further, the results showed the incremental value of Criterion A beyond the level of underlying psychopathology and maladaptive personality traits suggesting the distinctive function of Criterion A to capture the features of borderline personality. These findings extend the knowledge about the dimensional aspects of personality pathology in adolescence. The implications in relation to the new personality disorder model in the ICD-11 are highlighted.

## Introduction

During the last decade, the field of personality disorder (PD) research and practice has been moving to adopt a dimensional approach. The major classification systems—the publication of the Alternative Model for Personality Disorders (AMPD) in the 5th revision of the Diagnostic and Statistical Manual of Mental Disorders [DSM-5; (1)] and the 11th edition of the International Classification of Diseases [ICD-11; (2)] introduce a two-step dimensional conceptualization of personality pathology which emphasizes two different aspects that contribute to the maladaptive personality: the level of impairment in personality functioning and maladaptive personality traits.

In the AMPD model, the first component—Criterion A referred to as the Level of Personality Functioning (LPF)—defines deficits in self-functioning and interpersonal relatedness as a core and unidimensional severity mark of personality pathology. LPF includes disturbances of self-function in identity and self-direction domains and dysfunctions of empathy and intimacy as indicators of impaired interpersonal function. The second component of the dimensional model—Criterion B or maladaptive personality traits—is intended to represent a stylistic manifestation of PD by assessing five major domains of traits—namely, negative affectivity, detachment, antagonism, disinhibition, and psychoticism (3). These two constructs, required in operationalizing and determining PD, are separate facets of personality pathology (4). Whilst the diagnostic criteria A and B stem from distinct scholarly traditions (5, 6) and are intended to serve different functions in the dimensional model, a number of studies have demonstrated a considerable overlap between severity (Criterion A) and trait (Criterion B) ratings with traits accounting for considerable and incremental variance in personality impairments (7, 8). In a search for the unique role of both components in diagnosing PD, some research also revealed the added value of Criterion A over B in support of LPF as a severity measure of personality pathology and a unique predictor of specific PDs in adult samples (9).

Although adolescence is acknowledged to be a sensitive period for the development of personality disorder and the validity of the latter has been supported by numerous studies (10–12), empirical investigations evaluating Criterion A and B simultaneously, especially their interconnection during this period, lag behind those with adults (13). We think that research findings regarding the specificity of Criterion A and B for adult personality pathology cannot be directly transferred to the adolescent population when personality pathology is emerging (14). According to the theoretical integrated developmental view of personality pathology, Criterion A has been suggested to account for the onset of PD in adolescence, while Criterion B is observable before adolescence and reflects continuous aspects of maladaptive personality traits (15, 16). Thus, during adolescence, the manifestation and function of Criterion A are proposed to emerge (14). To date, the roles of Criterion A and B for personality pathology in adolescents have been examined separately (3, 17, 18). Namely, Goth et al. (17) developed a specifically AMPD tailored instrument—the Level of Personality Functioning Questionnaire [LoPF-Q 12–18]—to study Criterion A in adolescence and showed substantial differences between adolescents with and without PDs. Similarly, Weekers et al. (19) using the Semi-Structured Interview for Personality Functioning according to DSM-5 found that personality functioning impairment (Criterion A) is a sensitive indicator of personality pathology, especially borderline PD (BPD), which is the earliest to emerge in adolescence. Furthermore, empirical findings revealed disturbances in identity and self-direction (self-dysfunction) as well as intimacy (interpersonal dysfunction) to be the most prominent in adolescents with borderline personality pathology (17). As it comes to the second component, the developmental view of PD posits Criterion B as being already evident in childhood personality traits that continue into adolescence (16). Existing longitudinal evidence supports early maladaptive personality traits as an overall vulnerability factor for later PDs (20). For example, De Clercq et al. (21) findings suggested that children with a severe onset level of oddity-related characteristics were more at risk for developing personality pathology as described in the AMPD (based on compound scores of PID-5 maladaptive personality traits facets), especially schizotypal and borderline PDs. Another study showed that BPD can be predicted from childhood personality difficulties, with irritable-aggressive traits and affective lability being the core components (22). This briefly mentioned empirical evidence maps a trajectory of maladaptive traits (Criterion B) starting in childhood and continuing into adolescence (20). Taken together, while the studies of Criterion A and B suggest both being evident in adolescent personality pathology, their unique role is yet to be singled out, especially that of Criterion A. Beside this, a context of mental disorders should be considered as psychopathological symptoms have been established to be a risk factor for personality pathology (23), its severity (24), and course over adolescence (25).

Although Criterion A has been considered a core aspect for PDs, its interplay with maladaptive traits when investigating personality dysfunctions during adolescence has been scarcely studied so far (26, 27). Moreover, to our knowledge, no study to date has linked these two components in relation to adolescent personality pathology in general and to borderline personality features in particular. The change in the conceptualization of PD in both DSM-5 AMPD, as well as ICD-11, motivates understanding its link with categorically established BPD among adolescents which has been supported by extant research to date (11, 12, 20). So, a notable feature of the current study is that it is the first to examine the link between Criterion A and B and how they account for borderline features in a large sample of adolescents. We build our main hypothesis within the developmental framework of personality pathology (15, 16) by focusing on Criterion A to expect that it would be potent in predicting BP features among adolescents above and beyond the level of maladaptive personality traits and underlying (comorbid) psychopathological symptoms. Given a paucity of empirical findings related to the specificity of self and interpersonal dysfunctions, we had no specific hypothesis regarding their separate roles in predicting borderline features in adolescence. Further exploratory goals of the study were to shed more light on the interrelations of Criteria A and B as well as the association of Criterion B with borderline features among adolescents.

## Method

### Participants and Procedure

Participants were 568 adolescents aged 11–17 (*M* = 14.38, *SD* = 1.57; 42.4% males) recruited from public schools (*n* = 502; 40.6% males), a psychiatry inpatient unit (*n* = 41; 29.3% males), and a forensic unit for delinquent youth (*n* = 25; 100% males). Most adolescents were from urban areas (61.8%) and 33.5% were living in rural areas. Sixty percent of participants reported that their parents were married, 21%—divorced, and 19% indicated other family status.

Invitations to participate in the study along with informed written parent consent forms were distributed via schools, psychiatric and forensic adolescent care units. Adolescents who voluntarily agreed to participate in the study and whose parents gave written informed consent were asked to fill out the questionnaires. The study was administered by researchers or trained research assistants in small groups during school hours in the school sample and individually in both clinical and forensic samples. The study protocol was approved by the Psychological Research Ethics Committee at Vilnius University.

### Measures

The level of personality functioning (Criterion A) was assessed with the culturally adapted Lithuanian version of the Levels of Personality Functioning Questionnaire [LoPF-Q 12–18; (17, 28)]. It is a 97 item self-report instrument with a 5-step response format (0 = no to 4 = yes) with higher scores indicating a more severe level of impairment in personality functioning and a higher risk for a current personality disorder. The questionnaire allows to dimensionally assess the total score of personality dysfunction as well as adaptive function or disturbances in the self and interpersonal domains. The original questionnaire was developed by a research group in Basel University clinics, Switzerland. The adaptation procedure for the Lithuanian version of the LoPF-Q 12–18 (28) included the translation and back-translation of the items, the pilot, and main empirical studies to ensure the necessary psychometric qualities of the questionnaire. The main empirical study for the development of the Lithuanian version involved 362 adolescents (83% school-based sample; 17% clinical sample). The total score of the LoPF-Q 12–18 differentiated the subgroup of clinical adolescents (those with 5 or more BPD symptoms) from the school-based sample (Cohen's *d* = 1.2). The effect sizes on the subscale level were similar: identity (Cohen's *d* = 1.1), self-direction (Cohen's *d* = 1.1), empathy (Cohen's *d* = 0.5), and intimacy (Cohen's *d* = 1.0). The effect sizes of medium to large proved clinical validity of the LoPF-Q 12–18. In the current study, the internal consistency score was excellent for the total scale (α = 0.90). Cronbach's α on the subscale level was also high, accordingly identity (α = 0.90), self-direction (α = 0.94), empathy (α = 0.84), and intimacy (α = 0.87).

The short version of the Personality Inventory for DSM-5 for children aged 11–17 [PID-5-BF; (1)] was used to measure maladaptive personality traits (Criterion B). It comprises the 25 items rated on a 4-point scale (0 = very false to 3 = very true) and is categorized into 5 domains of maladaptive personality traits. A higher score indicates higher expression in the personality trait domain. To prepare the Lithuanian version of the PID-5-BF, two independent translations from English to Lithuanian were compared and the items were corrected to build the final version which was back-translated to English. The internal consistency was high for the total score (α = 0.91) and moderate for the following subscales: negative affectivity (α = 0.80), detachment (α = 0.70), antagonism (α = 0.68), disinhibition (α = 0.79), and psychoticism (α = 0.82).

The Borderline Personality Features Scale for Children [BPFSC-11; (29)] is an 11-item self-report questionnaire that was used to assess borderline personality features in adolescence. Participants' responses are rated on a 5-point Likert-type scale from “not true at all” to “always true” where higher scores indicate the higher expression of borderline features. The questionnaire captures the difficulties associated with emotional instability and interpersonal problems as core aspects of borderline personality disorder. In the inpatient sample of adolescents, BPFSC-11 performed well in identifying those who met the criteria for BPD according to the categorical approach to PD (29). To prepare the Lithuanian version of the BPFSC-11, two independent translations from English to Lithuanian were compared and the items were corrected to build the final version which was back-translated to English and approved by its authors (C. Sharp). In the current sample, Cronbach's α for the total scale was 0.88.

Youth Self-Report Form [YSR 11–18; (30)] was used to measure the level of psychopathological symptoms in adolescents. The total score is constituted of the items (*n* = 98) covering both the externalizing and internalizing spectrum difficulties, attention, social, thought, and other problems. The questionnaire has been fully adapted and standardized for use in the Lithuanian population (31). In this study, Cronbach's α for the total score of psychopathological symptoms was very high (α = 0.97).

### Statistical Analyses

Statistical Package for Social Sciences (SPSS) version 27 was used for statistical analyses (32). Testing the normality of the analyzed data demonstrated the sufficient normal distribution of all the questionnaires' scores on the total and subscale levels, with skewness and kurtosis values being in the range of −1 to 1 (except for antagonism which did not exceed 2). Thus, further analyses were conducted using parametric statistics. First, we computed descriptive statistics in the whole sample and its groups. Statistical significance of mean differences between groups was tested via one-way Analysis of Variance (ANOVA) and *post-hoc* tests. Next, we calculated the Pearson correlation coefficients to examine which dimensions of LoPF-Q 12–18 and PID-5-BF were related to the BPFSC-11 score. Finally, to examine the distinctive features of Criterion A, we explored a hierarchical linear regression model to test whether the level of personality functioning contributes to the prediction of borderline features when controlling for demographic variables (age and gender), psychopathological symptoms, and maladaptive personality traits.

## Results

Means and standard deviations for each subgroup (school, inpatient, and forensic) and the full sample are presented in Table 1. One-way ANOVA revealed significant differences between groups regarding the values of LoPF-Q 12–18 [*F*_(2,531)_ = 10.66, *p* < 0.01], PID-5-BF [*F*_(2,508)_ = 5.99, *p* < 0.01], and BPFSC-11 [*F*_(2,529)_ = 5.83, *p* < 0.01]. *Post-hoc* analyses (Bonferroni or Games-Howell) were conducted depending on the estimated equality of the variance in each subscale. Psychiatric inpatients were characterized by the most severe disruptions in personality functioning as well as the highest levels of maladaptive and borderline personality traits when compared to the forensic and school-based groups. Next, bivariate associations analysis using Pearson correlation coefficients (Table 2) showed that gender in the total sample significantly correlated with LoPF-Q 12–18 (*r* = −0.20, *p* < 0.01), PID-5-BF (*r* = −0.22, *p* < 0.01), and BPFSC-11 (*r* = −0.27, *p* < 0.01) scores such that girls had more disrupted personality functioning and presented more maladaptive personality traits and borderline features than boys. Also, older age was positively related to higher scores on PID-5-BF (*r* = 0.14, *p* < 0.01) and BPFSC-11 (*r* = 0.14, *p* < 0.01). Further correlational analysis revealed strong associations of BPFSC-11 with total scores of LoPF-Q 12–18 (*r* = 0.75, *p* < 0.01) and PID-5-BF (*r* = 0.80, *p* < 0.01) indicating that higher levels of disruptions in personality functioning or more prominent maladaptive personality traits were associated with higher levels of borderline features. Bivariate relations between Criterion A (LoPF-Q 12–18 total score and subscales) and Criterion B (PID-5-BF total score and subscales) had a robust pattern, with moderate to large in magnitude (see Table 2).

**Table 1:** Descriptive statistics by group for observed variables.

	**Score interval**	**School (*n* = 467)^a^**	**Inpatient (*n* = 40)^b^**	**Forensic (*n* = 25)^c^**	**Whole group (*n* = 568)**	***F***
		***M* (*SD*)**	***M* (*SD*)**	***M* (*SD*)**	***M* (*SD*)**	
BPFSC-11	11–55	28.87 (9.30)^b^	34.00 (9.05)^a^	30.44 (9.00)	29.33 (9.35)	5.83^**^
LoPF-Q total score	0–388	140.07 (59.78)^b^	184.99 (64.93)^a^, ^c^	143.32 (48.72)^b^	143.67 (60.80)	10.66^***^
LoPF-Q identity	0–92	34.27 (17.02)^b^	48.73 (18.84)^a^, ^c^	34.35 (11.27)^b^	35.38 (17.35)	13.77^***^
LoPF-Q self-direction	0–100	39.10 (21.17)^b^	58.96 (23.50)^a^, ^c^	37.32 (21.57)^b^	40.52 (21.98)	16.60^***^
LoPF-Q empathy	0–104	33.52 (14.22)	36.66 (15.06)	39.20 (12.79)	34.02 (14.27)	2.66
LoPF-Q intimacy	0–92	32.49 (14.98)^b^	40.63 (15.93)^a^, ^c^	32.45 (10.03)^b^	33.10 (14.50)	5.69^**^
PID-5-BF total score	0–75	24.25 (13.92)^b^	32.20 (13.73)^a^	25.90 (15.75)	24.94 (14.11)	5.99^**^
PID-5-BF negative affectivity	0–15	6.10 (4.03)^b^	8.35 (4.33)^a^, ^c^	5.04 (3.98)^b^	6.22 (4.09)	6.76^**^
PID-5-BF detachment	0–15	4.58 (3.28)	5.76 (3.56)	3.87 (3.25)	4.64 (3.31)	3.04^*^
PID-5-BF antagonism	0–15	2.89 (2.76)	3.17 (2.70)	3.92 (3.95)	2.96 (2.83)	1.69
PID-5-BF disinhibition	0–15	4.85 (3.52)^b^, ^c^	7.22 (3.37)^a^	6.83 (3.69)^a^	5.12 (3.59)	11.41^***^
PID-5-BF psychoticism	0–15	5.83 (3.99)^b^	8.12 (4.37)^a^, ^c^	5.24 (4.05)^b^	5.98 (4.06)	6.58^**^
YSR 11–18 total score	0–196	48.94 (31.77)^b^, ^c^	81.02 (39.23)^a^	70.32 (33.72)^a^	52.63 (33.87)	22.12^***^

* *p < 0.001*,

** *p < 0.01, and*

*** *p < 0.001*.

a, b, c *Significant differences between groups*.

**Table 2:** Correlations among study variables.

	**1**	**2**	**3**	**4**	**5**	**6**	**7**	**8**	**9**	**10**	**11**	**12**
BPFSC-11	–											
LoPF-Q total score	0.75											
LoPF-Q identity	0.73	0.92										
LoPF-Q self-direction	0.79	0.93	0.85									
LoPF-Q empathy	0.56	0.81	0.61	0.64								
LoPF-Q intimacy	0.52	0.87	0.74	0.71	0.68							
PID-5-BF total score	0.81	0.80	0.74	0.77	0.67	0.62						
PID-5-BF negative affectivity	0.74	0.63	0.63	0.69	0.45	0.40	0.81					
PID-5-BF detachment	0.52	0.67	0.59	0.59	0.54	0.67	0.78	0.48				
PID-5-BF antagonism	0.42	0.42	0.33	0.34	0.55	0.30	0.61	0.37	0.40			
PID-5-BF disinhibition	0.67	0.67	0.64	0.64	0.55	0.50	0.83	0.60	0.59	0.40		
PID-5-BF psychoticism	0.72	0.69	0.63	0.68	0.55	0.56	0.86	0.63	0.60	0.41	0.63	
YSR 11–18 total score	0.64	0.71	0.66	0.70	0.56	0.52	0.67	0.56	0.47	0.40	0.57	0.58

*All values are significant at p < 0.001*.

At the final step, a hierarchical linear regression model was tested to analyze the variance accounted by Criteria A and B on borderline personality features in the studied sample. The examination of multicollinearity revealed that variance inflation factor (VIF) for all variables was not larger than 5.37 (LoPF-Q 12–18 self-direction subscale) and tolerance values were not smaller than 0.19 (LoPF-Q 12–18 self-direction subscale). It is suggested that VIF values not larger than 10 (33) and tolerance values not smaller than 0.10 (34) are not indicative of problematic multicollinearity, so we proceeded with further analysis. In this model BPFSC-11 score was regressed on age, gender (Step 1), total problems score of YSR 11–18 (Step 2), following PID-5-BF five trait domains (Step 3), and LoPF-Q 12–18 four functioning dimensions (Step 4).

The results of regression analysis are presented in Table 3. It was found that PID-5-BF domains captured a significant amount of unique variance (25.6%) in the prediction of the BPFSC-11 scores when controlling for age, gender, and total score of psychopathological symptoms (Step 3). At this step, negative affectivity (β = 0.32, *p* < 0.01), disinhibition (β = 0.20, *p* < 0.01), and psychoticism (β = 0.27, *p* < 0.01) along with total score of YSR (β = 0.19, *p* < 0.01) were significant predictors. A few interesting findings emerged in Step 4. First, the LoPF-Q 12–18 domains incrementally contributed an additional 4.2% of the variance. In detail, identity (β = 0.10, *p* < 0.05), self-direction (β = 0.33, *p* < 0.01), and intimacy (β = −0.10, *p* < 0.05) were statistically significantly associated with borderline personality features. Second, an unexpected finding here has been the change in the direction of association between LoPF-Q 12–18 intimacy domain (LoPF-Q 12–18) and borderline personality features from positive zero-order correlation into negative beta weight. This indicates a manifestation of negative statistical suppression in which the relationship between a predictor and the outcome variable reverses after adjusting for additional predictors (35). The suppression has likely appeared because of strong correlations of the intimacy domain with other predictors and the dependent variable (BPFSC-11). When entered into the regression equation Intimacy subscale increased the predictive power of other predictors by removing irrelevant variance from them and gaining negative weight. Third, the association between borderline features and psychopathological symptoms was no longer significant at this step (Step 4) when controlling for Criterion A domains. However, negative affectivity (β = 0.22, *p* < 0.01), disinhibition (β = 0.13, *p* < 0.01), and psychoticism (β = 0.21, *p* < 0.01) continued to be statistically significant predictors.

**Table 3:** Hierarchical linear regression analysis for predicting BPFSC-11 scores.

**Predictor variables**	***B***	**SE**	**Beta**	***t***	***p***	***R*** **^2^**	***R*** **^2^ change**	***F***
**Step 1**						0.10	0.10	23.92^*^
Age	0.92	0.27	0.16	3.44	0.00			
Gender	−5.38	0.85	−0.28	−6.30	0.00			
**Step 2**						0.44	0.35	282.34^*^
Age	−0.09	0.22	−0.02	−0.43	0.67			
Gender	−2.80	0.69	−0.15	−4.08	0.00			
YSR 11–18 total problems	0.17	0.01	0.63	16.80	0.00			
**Step 3**						0.70	0.26	75.80^*^
Age	−0.19	0.16	−0.03	−1.16	0.25			
Gender	−1.08	0.54	−0.06	−2.01	0.05			
YSR 11–18 total problems	0.05	0.01	0.19	5.25	0.00			
PID-5-BF negative affectivity	0.74	0.09	0.32	8.46	0.00			
PID-5-BF detachment	−0.07	0.10	−0.03	−0.73	0.47			
PID-5-BF antagonism	0.15	0.10	0.04	1.47	0.14			
PID-5-BF disinhibition	0.52	0.10	0.20	5.17	0.00			
PID-5-BF psychoticism	0.60	0.09	0.27	6.61	0.00			
**Step 4**						0.74	0.04	18.08^*^
Age	−0.03	0.15	0.00	−0.18	0.85			
Gender	−0.46	0.52	−0.02	−0.90	0.37			
YSR 11–18 total problems	0.01	0.01	0.05	1.34	0.18			
PID-5-BF negative affectivity	0.51	0.09	0.22	5.87	0.00			
PID-5-BF detachment	−0.14	0.10	−0.05	−1.33	0.18			
PID-5-BF antagonism	0.20	0.10	0.06	1.89	0.06			
PID-5-BF disinhibition	0.34	0.10	0.13	3.47	0.00			
PID-5-BF psychoticism	0.48	0.09	0.21	5.55	0.00			
LoPF-Q identity	0.06	0.03	0.10	2.01	0.04			
LoPF-Q self-direction	0.14	0.02	0.33	5.92	0.00			
LoPF-Q empathy	0.02	0.03	0.03	0.84	0.40			
LoPF-Q intimacy	−0.06	0.03	−0.10	−2.28	0.02			

* *p < 0.05*.

## Discussion

The current study aimed to analyze the associations of Criterion A and B—the components of the contemporary dimensional model of personality disorder—with borderline personality features among adolescents. In line with the described developmental trajectory of personality pathology in adolescence (16), we were particularly interested in the unique role of Criterion A to account for borderline personality features after adjusting for the maladaptive personality traits (as defined in Criterion B) and underlying psychopathological symptoms. To examine this, we used a large sample covering a spectrum from typical to problematic development (school-based sample, psychiatric inpatients, and delinquent youth) and a broad adolescence age span along with the measure of LPF—LoPF-Q 12–18—specifically developed for adolescents under the frame of the AMPD in DSM-5 and entry criterion for PDs diagnostic model in ICD-11 (17).

Several findings emerge from this study. First, consistent with our main hypothesis, the findings of the present study suggest the importance of Criterion A for borderline personality features in adolescents. Specifically, the results of our regression model showed the statistically significant unique association between Criterion A and borderline features beyond the context of underlying psychopathology and maladaptive personality traits. This allows us to maintain and strengthen the arguments that Criterion A should have its distinctive function in capturing the features of adolescent personality pathology (15, 36). Research with adults has already shown that personality dysfunction taps a core of personality disorder (37), its specific aspects (7, 38), or outcomes (39). The results of our study extend at least some of these findings into the period of adolescence by pointing to the necessity to consider the level of personality functioning in understanding early borderline personality features. This is particularly important with regard to the new ICD-11 approach which bases assessments of PD on a patient's personality functioning. Accordingly, such dysfunction should also explain the borderline pattern qualifier traditionally called BPD (2). Our findings confirm that this approach is essential in evaluating personality pathology in adolescence too. Furthermore, results from the present study support that the self-functions—identity and self-direction—contribute significantly to the variance of borderline features among adolescents. However, the presence of statistical suppression found in our study doesn't allow us to interpret the role of intimacy in the understanding of borderline features when these are explained simultaneously using other variables of the study. Although the likelihood of suppressor effects can be attributed to a mere statistical artifact (35), it may also be a replicable phenomenon as has been the case in other research fields, e.g., personality traits (40), coping (41), or developmental links between anxiety and depression (42). Our results point at the need for further elaboration on the association of the LoPF-Q 12–18 with borderline personality features. In another sample of Lithuanian adolescents (*N* = 362, unpublished data available from the first author upon a request) the same type of statistical suppression appears. It is not clear yet it is a culture-specific or a general phenomenon, but it waits to be tested in other populations.

Next, the regression model revealed further that Criterion B domains retained their significance when predicting borderline personality features together with Criterion A dimensions. As of note, negative affectivity is postulated to be the most consistent correlate of borderline pathology, along with disinhibition and antagonism (43–45). Differently than explained, the results of the current study revealed a significant contribution of psychoticism which along with negative affectivity had the strongest correlations with, and in conjunction with disinhibition explained the variance of borderline personality features. Although the association of negative affectivity and disinhibition with borderline pathology is in line with the dimensional model of BPD, psychoticism is not among its diagnostic criteria in DSM-5 (1). Nevertheless, psychoticism has been found to map borderline pathology in adults in terms of cognitive and perceptual dysregulation, including proneness to dissociation (46, 47). Notable, the ICD-11 captures such reality testing features in terms of global severity thus aligning them with functioning (1, 48, 49). In other studies, psychoticism has been found to overlap with internalizing and externalizing components that mark a general tendency of dysfunction in young individuals (50).

To the best of our knowledge, the present study is the first to shed light on the functions of Criteria A and B relative to personality disturbances among adolescents. Overall, it provides evidence that both criteria supplement in indicating borderline personality features in adolescence and might benefit from aspects of one another. These two aspects of the dimensional model—Criterion A, as measured by the LoPF-Q 12–18, and Criterion B, as measured by the PID-5-BF—were highly interrelated in the current study. The associations between Criterion A and B might be anchored and interpreted from a developmental perspective on personality pathology (20). The recent study evidenced a longitudinal prediction of personality traits on personality (self)functioning over the period of 10 years (51). Thus, the cross-sectional interconnection between Criterion A and B could also mark the potential contribution of maladaptive traits to personality dysfunction.

Overall, the findings of our study endorse the relevance of the dimensional model to capture (borderline) personality problems during adolescence. The level of personality functioning is a necessary entry criterion for PD diagnostics in both classification systems—DSM-5 (1) and ICD-11 (2). For the latter, it is the only one required. The present study can shed some light on the implications for ICD-11. First, it reaffirms that BPD in adolescence is a matter of personality functioning, just as studies with adults have shown: rather than being distinct psychopathology, BPD is the strongest marker of the general PD factor (52) and “disappears” into it (37). As such, understanding borderline PD once again brings us closer to the level of personality organization as defined by Kernberg (53) and suggests that BPD criteria reflect the core features of PD severity (37, 54). Secondly, the retention of the borderline qualifier in the ICD-11 raises the question of its possible redundancy with the PD severity criterion (54). The high correlations between personality functioning, maladaptive traits, and borderline features found in the current study suggest that it is a relevant question in adolescence too. Finally, the use of ICD-11 requires assessment tools. Some studies have shown that measures originally developed for Criterion A in the AMPD can be reliably used to classify the severity of PD in the ICD-11 (55). In light of these results, the operationalization of personality functioning used in the current study, the Level of Personality Functioning Questionnaire for adolescents (LoPF-Q 12–18), uniquely captures adolescents' (borderline) personality difficulties (17, 18), and might be considered a proxy measure for PD severity in the ICD-11.

Despite these contributions, the current results are subject to several limitations. First, as the study included only self-report measures only, this could lead to method-inherent pitfalls in each sample. Empirical studies have shown that self-report scores on personality functioning should be interpreted cautiously in forensic settings (56). Secondly, it used a specific measure of BPFSC-11 which limits the results to the current measure of borderline personality. Third, although we used a large sample of adolescents inclusive of clinical and risk groups to maximize the variance in the assessed outcome, studies with larger clinical samples are needed. Fourth, other criterion variables, e.g., psychosocial functioning might help to shed light on the further delineation of the specificity and difference in functions of Criterion A and B as it has been shown in the studies with adults (38). Finally, the study employed the cross-sectional, not longitudinal design which as we note in the above text could specify better the value of Criterion A and B in relation to personality pathology during adolescence as a sensitive period (36).

In sum, the current research provides an important step in understanding how the main components of the dimensional model work together to indicate and describe borderline personality features that are the earliest maladaptive personality indicator to emerge in development (19).

## Data Availability Statement

The raw data supporting the conclusions of this article will be made available by the authors, without undue reservation.

## Ethics Statement

The studies involving human participants were reviewed and approved by Vilnius University Psychological Research Ethics Committee. Written informed consent to participate in this study was provided by the participants' legal guardian/next of kin.

## Author Contributions

RB: conceptualization, data analysis, and writing the initial draft. EG: data collection, contribution to the introduction section of the paper, and writing. AA: data collection and writing. LG-P: contribution to the results section of the paper and writing. AL: data collection, contribution to the data analysis, results, discussion sections of the paper, and writing. GS-N: data collection and curation, contribution to the data analysis, methods, results sections of the paper, and writing. All authors contributed to the article and approved the submitted version.

## Funding

This study was funded by a grant (No. S-MIP-21-20) from the Research Council of Lithuania. Open access publication fees were funded by Vilnius University.

## Conflict of Interest

The authors declare that the research was conducted in the absence of any commercial or financial relationships that could be construed as a potential conflict of interest.

## Publisher's Note

All claims expressed in this article are solely those of the authors and do not necessarily represent those of their affiliated organizations, or those of the publisher, the editors and the reviewers. Any product that may be evaluated in this article, or claim that may be made by its manufacturer, is not guaranteed or endorsed by the publisher.
